# What Is the Trait d’Union between Retroactivity and Molecular Communication Performance Limits?

**DOI:** 10.3390/molecules27103130

**Published:** 2022-05-13

**Authors:** Francesca Ratti, Maurizio Magarini, Domitilla Del Vecchio

**Affiliations:** 1 Mechanical Engineering Department, Massachusetts Institute of Technology, Cambridge, MA 02139, USA; ddv@mit.edu; 2 Department of Information, Electronics, and Bioengineering, Politecnico di Milano, 20133 Milan, Italy; maurizio.magarini@polimi.it

**Keywords:** molecular communication, mutual information, systems biology, synthetic biology, communication systems, retroactivity

## Abstract

Information exchange is a critical process in all communication systems, including biological ones. Retroactivity describes the load that downstream modules apply to their upstream systems in biological circuits. The motivation behind this work is that of integrating retroactivity, a concept proper of biochemical circuits, with the metrics defined in Information Theory and Digital Communications. This paper focuses on studying the impact of retroactivity on different biological signaling system models, which present analogies with well-known telecommunication systems. The mathematical analysis is performed both in the high and low molecular counts regime, by mean of the Chemical Master Equation and the Linear Noise Approximation, respectively. The main goal of this work is to provide analytical tools to maximize the reliable information exchange across different biomolecular circuit models. Results highlight how, in general, retroactivity harms communication performance. This negative effect can be mitigated by adding to the signaling circuit an independent upstream system that connects with the same pool of downstream circuits.

## 1. Introduction

Molecular Communication (MC) is a field of research that has gained relevance in recent years. This emerging discipline is directly inspired by natural communications between cells in biology [1,2]. Characterizing living cells from an information and communication theoretical perspective is one of the keys to understand the fundamentals of MC system engineering [3]. In parallel with the experimental applications of MC [4,5,6], the development of mathematical models for many of the phenomena occurring in an MC system has gained notice lately. There exist several types of MC channels, e.g., single-cell, multiple-cell, and cell-population [7]. From a modeling perspective, great attention has been paid to the methods of propagation of molecules in the extracellular environment [8,9,10,11,12]. A step forward has been that of studying the communication performance in biochemical circuits [13,14]. In fact, an open research problem in MC is that of finding optimal ways to transfer information in biochemical circuits [15]. Several works have investigated this problem recently. For example, in [16] the authors analyze the channel capacity of a biological system considering both diffusion-based channel and ligand-based receiver. Moreover, in [17] the authors provide a closed-form expression for the information capacity of an MC system with a noisy channel. In [18], a different approach is taken, where the authors use enzymatic reaction cycles to improve the upper bound on the mutual information (MI) for a diffusion-based MC system. The work in [19] presents an analysis of the channel capacity in diffusive MC by considering intersymbol interference from all the previous time slots and the channel transmission probability in each time slot.

Furthermore, there exist several works in the literature investigating parallelisms between well-known telecommunication and MC models and evaluating information exchange performance in different biological scenarios. For example, in [20] the authors focus on the interference that is generated in the case of a broadcast channel, i.e., when the same transmitter (e.g., a cell) sends the same message simultaneously to multiple receivers (e.g., several cells). In [21], the authors present and develop models for the molecular multiple-access, broadcast, and relay channels in a MC system and perform a numerical analysis on their capacity expressions. The authors of [22] study the capacity of a multiple-access channel that is affected by the parameters of the diffusive channel and ligand-receptor binding mechanisms. The work in [23] presents a training-based channel impulse response estimation for diffusive *multiple-input multiple-output (MIMO)* channels. In [24], a MIMO design for MC is proposed, where multiple molecular emitters are used at the transmitter and multiple molecular detectors are used at the receiver. Various diversity techniques for MIMO transmissions based on molecular diffusion are proposed in [25] to improve the communication performance in nanonetworks in the presence of multi-user interference.

The common thread among the aforementioned works is the focus on the communication in the extracellular environment, where propagation of molecules plays a fundamental role in the communication performance. In this paper, we concentrate on biomolecular circuits whose communication is not prominently affected by propagation, and, in particular, we focus on isolating the impact of *retroactivity* on the communication performance. Retroactivity is the effect that downstream systems receiving a signal apply to upstream ones sending the signal. It can be seen as an extension of the concept of impedance of electrical circuits to biomolecular systems because the additional binding/unbinding reaction which constitutes the downstream system competes with the biochemical interactions constituting the upstream system. Thus, it may disrupt the operation of the upstream system [26].

Since its introduction in the literature [26], retroactivity has been studied in the contexts of control theory and systems biology, with the aim of characterizing and efficiently designing modular biomolecular circuits [27,28,29,30,31,32,33,34,35]. The back-propagated signal generated by the interconnected components plays a role not only in the design of biomolecular circuits but also in the communication performance of the upstream system.

There exist a few works in the literature considering the effects of retroactivity on the information exchange in molecular circuits. The author of [36] presents preliminary results on the impact of retroactivity on the communication performance of a diffusive MC system, composed of one transmitter and one receiver. A review of retroactivity in different signaling systems and genetic circuits can be found in [37].

The main contribution of this paper consists of the analytical investigation of the impact of retroactivity on the information exchange for different MC system models. This is achieved by making parallelisms with some well-known telecommunication ones. The ultimate objective is to lay foundations of how to efficiently maximize the reliable information exchange when considering different signaling circuits. In the following, we illustrate the results we achieved in this direction. Preliminary outcomes were presented in [38].

The paper is organized as follows. Section 2 provides a high-level summary of the work, by introducing its motivations and by presenting a visual scheme resuming the main steps. In Section 3, we introduce the theoretical concepts on which our work lays its foundations, and we contextualize them by explaining the preliminary assumptions we made. Then, in Section 4, we present the biochemical system models on which we perform the communication performance evaluation, by making parallelisms with well-known telecommunication models. In Section 5, we present and discuss the analytical results. Last, in Section 6 we conclude the paper.

## 2. Overview

The fields of MC and of systems and synthetic biology have as a final goal that of understanding biological phenomena, and that of “engineering” them to improve upon their nature when possible or necessary, e.g., to combat diseases [39,40], or to enhance agricultural processes [41,42]. We asked ourselves if the way a biological system is built can affect its communication performance, and, in particular, *if and how retroactivity affects MC communication performance.* In fact, to the best of our knowledge, this question has not been answered in the literature yet. Awareness regarding the effect of retroactivity on communication performance is relevant both in the context of understanding and maximizing the information flow [43,44] and in that of designing biological circuits [45,46]. The goal of this paper is that of filling this gap.

**Figure 1:**
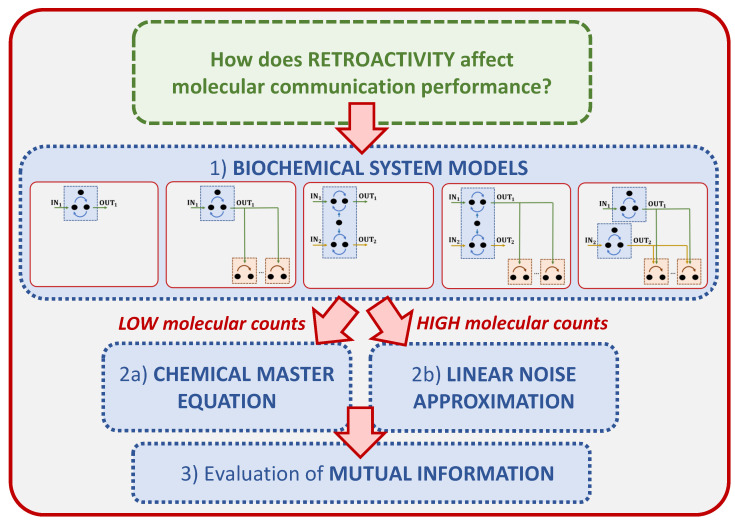
Overview of the structure of the paper. The biochemical system models are detailed later in Figure 3. The Chemical Master Equation (CME) is a system of differential equations that describes the rate of change of the probability of the system to be in any give state at time *t* [47]. The Linear Noise Approximation (LNA) of the CME is a linear time-varying stochastic differential equation that allows a stochastic characterization of the evolution of a chemical reaction network, still maintaining scalability comparable to that of the deterministic models [48].

Figure 1 visually summarizes the main steps we have taken. We consider five biochemical systems, which enable us to quantify the effect of retroactivity in a variety of scenarios. All of them present similarities with most of the common digital communication models. These will be detailed in Section 4.

We model the stochastic behavior of each biochemical system both in the low molecular counts regime via the Chemical Master Equation (CME) [47], and in the high molecular counts regime via the Linear Noise Approximation (LNA) [48]. Although the CME can describe systems in high molecular count regimes, its analysis in these cases is computationally demanding. Therefore, supposing that the biochemical reaction rates are time-invariant, we opted for using the LNA in the high molecular count regime. In this way, we can compute a tractable analysis for the simpler models, and to make some speculations on the more complex ones. Both the CME and the LNA are solved at *steady state*. In fact, we consider the symbol completely received when the system reaches steady state, so that we avoid the effects of the transient behavior of the biomolecular circuit on the communication exchange performance, and we are able to isolate the contribution of the retroactivity. Please note that when dealing with non-equilibrium systems, steady state solutions may not be achieved. This would imply the necessity to compute the solution of the CME and of the LNA with respect to time, thus the evaluation of the MI in time. In this work we suppose the steady state solution of the considered systems to exist. The CME and LNA allow determining the probability mass functions (pmf) and the probability density functions (pdf), respectively, necessary for the evaluation of the MI between the input and the output of the system.

## 3. Preliminaries

The core of this work lays its foundations on concepts coming from different fields, i.e., digital communication, information and control theory, and biology. In this section, we introduce the main theoretical background of this study.

### 3.1. Retroactivity

Each chemical reaction that, starting from a compound, produces another molecule can be regarded as an input/output system. Such an input/output system can, in turn, be regarded as a module that, when connected with others, forms a more complex system. A fundamental issue that arises when interconnecting different components is how the process of transmitting a signal to a “downstream” module affects the dynamic state of the “upstream” subsystem sending the signal. In fact, on interconnection, a signal that goes from the downstream to the upstream system is generated. This phenomenon is called *retroactivity*, formally defined as the back action from the downstream system to the upstream one [26].

Retroactivity conceptually differentiates from feedback. In fact, *while it is not possible to transmit the output of the upstream system to the downstream one without retroactivity, on the contrary, feedback from the downstream system can be eliminated even when the upstream system is transmitting the signal.* As previously mentioned, while the impact of retroactivity on the design of biomolecular circuit has already been investigated, and solutions to mitigate this effect have been presented, the effects of retroactivity on the communication performance have not been analyzed in depth in the literature.

In our paper, we consider different models of signaling circuits. These are molecular circuits that, given external stimuli (inputs), through a series of chemical reactions, transform them to signals (outputs) that can control how cells respond to their environment. Then, we analyze to what extent retroactivity from downstream systems affects the communication performance between the input and the output of the upstream system.

**Figure 2:**
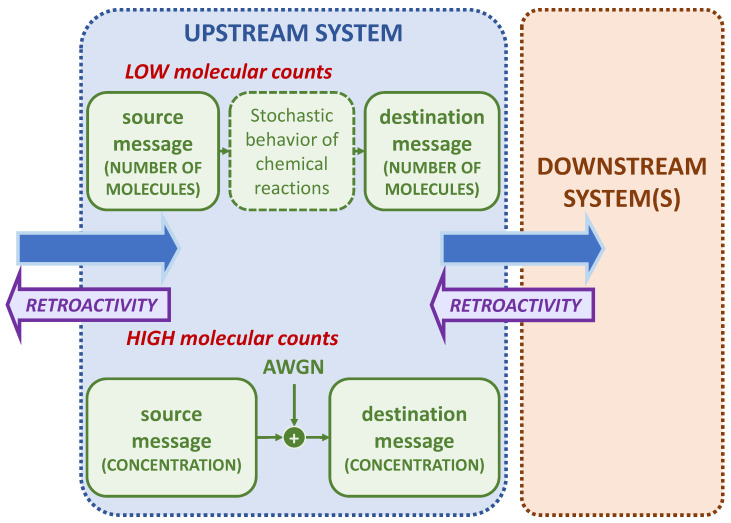
Upstream signaling system in the case of low and high molecular counts. The interconnection between the upstream and the downstream system generates the signal going from right to left in the figure (mauve arrow), that is the effect of retroactivity. The blue arrow from left to right represents the signal transmitted throughout the system.

Figure 2 shows a general scheme of signal exchange between upstream and downstream systems. The blue arrows represent the signal passing through the input of the upstream system to the downstream system. The mauve arrows represent the effect of retroactivity, i.e., the signal that goes from the downstream to the upstream system. We will perform the analysis for both the low and high molecular count regimes. In the figure, the upstream system describes the communication models representing both scenarios, which are detailed in the next sections.

### 3.2. Stochastic Models for Biochemical Systems

The evaluation of the MI as difference of entropies [49] requires the knowledge of the marginal pmfs (pdfs in the continuous case) $P\left(X\right)$, $P\left(Y\right)$, and the conditional pmfs $P\left(Y|X\right)$, $P\left(X|Y\right)$. Thus, a stochastic modeling to approximate the behavior of the considered systems at steady state is needed. Several modelings have been proposed in the literature [48,50,51,52,53]. As anticipated in Section 2, in this paper we model the behavior of the system in the low molecular count regime via the CME [47,51], while we use the LNA [48] under the assumption of high molecular counts.

#### 3.2.1. The Chemical Master Equation

The CME describes the rate of change of the probability of a molecular microstate in a chemical reaction system [47]. For a system with N chemical species, a microstate represents the number of molecules present for each species in the system at a given time. Thus, it requires the enumeration of all the possible microstates of the system (i.e., each possible microscopic configuration). In this work, as previously mentioned, we consider steady state, i.e., we set to zero the rate of change of the probability in the CME.

The motivations behind the choice of the CME to model the behavior of the system are two. First, we consider the accuracy of the results worth the complexity of the calculations. Furthermore, the CME does not take into account any stochastic effects other than the ones due to intrinsic noise in the chemical reactions. In this way, we can selectively quantify the effect of retroactivity on information exchange for different biomolecular system models. The major drawback of the CME is the computational complexity in computing the probability of being in each microstate at time *t*, which increases exponentially as the number of molecules and reactions in the system increases. For this reason, we use the CME only in the low molecular counts regime, and we rely on the LNA for studying the behavior of systems with a higher number of molecules.

#### 3.2.2. The Linear Noise Approximation

The LNA lays its foundations on the central limit theorem from probability theory [54], which states that, when independent random variables are added, their properly normalized sum tends toward a normal distribution, even if the original variables themselves are not normally distributed. This is why the LNA is valid only in the case of high molecular counts. Thus, when applying the LNA to model the stochastic behavior of a biochemical circuit, the resulting distributions of the species concentrations of the system in time are Gaussian. When using the LNA, the channel noise is assumed Additive White Gaussian (AWGN), with variance $\sigma_{noise}^{2}=1$, hence simplifying the communication model. The mean of the distribution is obtained by the reaction rate equations of the system [47]. In our case, since we work at steady state, these differentials equations are solved for the equilibrium. The steady state covariance matrix of the system is computed by solving the Lyapunov Equation [47]. As it can be noticed in the upstream system of Figure 2, in the case of the LNA model, the unit of measure of the biochemical species is concentration rather than number of molecules as in the CME. Thus, the number of molecules in the system is to be divided by the reaction volume.

## 4. System Model

In this section, we present the chemical reaction models analyzed in the paper. The chemical reactions are divided into subgroups representing the upstream and downstream systems. The addition of complex and realistic phenomena, e.g., particle movement dynamics, intersymbol interference, and memory is out of the scope of this work. Retroactivity is an effect present in all biocommunication systems, and the goal is to analyze and isolate its impact on MI. The construction of a realistic biological scenario implies the introduction of many factors in the evaluation of the MI, making unnecessarily difficult the isolation of the impact of retroactivity in this context.

**Figure 3:**
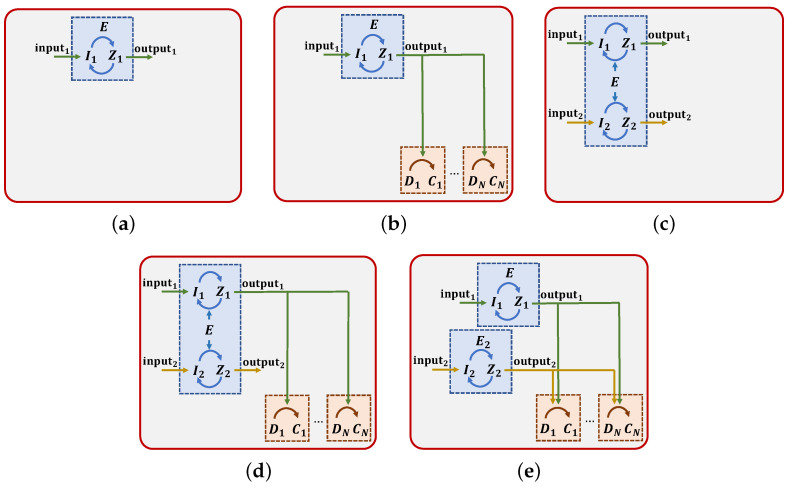
Diagrams of the signaling systems analyzed in the paper: (**a**) isolated SISO, (**b**) SISO with *N* downstream targets, (**c**) isolated MIMO, (**d**) MIMO with *N* downstream targets, (**e**) two isolated SISO with MAC.

More specifically, we consider the five signaling system models shown in Figure 3. Figure 3a represents an isolated signaling system, which is not connected to any downstream target. In digital communication, this could be viewed as a *Single-Input Single-Output (SISO)* system, where the input is $I_{1}$, and the consequent output is $Z_{1}$. Then, in Figure 3b, the same isolated system is connected through $Z_{1}$ to *N* chemical circuits. Thus, in Figure 3b, the system of Figure 3a becomes an upstream system directly linked to *N* downstream ones. This model could be viewed as a *Broadcast channel (BC)*, where the output of the upstream system $Z_{1}$ is the same message sent to different receivers, i.e., the downstream systems. The third signaling system (Figure 3c) models the same upstream system in the presence of a second circuit that shares the same enzyme E. We model the second circuit as a replica of the first one. This recalls the *MIMO* setup typical of the telecommunication systems, where the multiple inputs are $I_{1}$ and $I_{2}$ and the outputs are $Z_{1}$ and $Z_{2}$, respectively. The fourth considered signaling system is illustrated in Figure 3d. It is a MIMO model in which the output of the first upstream system $Z_{1}$ is connected to *N* downstream targets. The last model considers two isolated SISO upstream systems that can connect to the same *N* downstream systems. Please note that only one of the two upstream systems can connect with a specific downstream system at a time. This means that when both upstream systems are sending information, the number of downstream systems available to receive the message $Z_{1}$ is Q≤N. This recalls the *Multiple-Access Channel (MAC)* of digital communication, where the two upstream systems are the different transmitters that do not coordinate, and the downstream systems are the receivers that communicate with both the transmitters.

The two-step enzymatic reaction representing the upstream system can be described as

$$\begin{array}{c} I_{1}+E\overset{k_{0}}{⇌}M_{1}\\ M_{1}\overset{\;c_{1}\;}{\to }E+Z_{1}\\ Z_{1}\overset{\;c_{2}\;}{\to }I_{1}, \end{array}$$

with conservation laws $E_{\mathrm{tot}}=E+M_{1}$, and $I_{{\mathrm{tot}}_{1}}=I_{1}+M_{1}+Z_{1}$, where $I_{1}$ is the protein (input message) that binds to an enzyme E to form the complex $M_{1}$, that in turn is transformed in the output protein $Z_{1}$ [47]. The coefficients $c_{1}$ and $c_{2}$ are the catalytic rates of the unidirectional chemical reactions, and $k_{0}=k_{0}^{-}\;/\;k_{0}^{+}$ is the so-called dissociation constant of the reversible binding reaction, $k_{0}^{+}$ and $k_{0}^{-}$ being the association and dissociation rate constants [47]. The last reaction is represented by a one-step rather than a two-step enzymatic reaction to reduce the complexity of the calculations in the following. Furthermore, note that this last reaction makes the system cyclic. This is a major discrepancy with respect to traditional communication models, which are unidirectional. The secondary upstream circuit in the last three models is composed of an analogous two-step enzymatic reaction, with input $I_{2}$ and output $Z_{2}$, with conservation law $I_{{\mathrm{tot}}_{2}}=I_{2}+M_{2}+Z_{2}$. Thus, the conservation law that preserves the total amount of enzyme present in the system becomes $E_{\mathrm{tot}}=E+M_{1}+M_{2}$ for the models in Figure 3c,d. The model in Figure 3e is characterized by two separate conservation laws that preserve the quantity of the enzymes E and $E_{2}$, that are, respectively, $E_{{\mathrm{tot}}_{1}}=E+M_{1}$ and $E_{{\mathrm{tot}}_{2}}=E_{2}+M_{2}$.

Each *j*th downstream system is composed of one reversible reaction

$$Z_{1}+D_{j}\overset{k_{3_{j}}}{⇌}C_{j}$$

with conservation law $D_{{\mathrm{tot}}_{j}}=D_{j}+C_{j}$ Here, the output of the upstream system Z${}_{1}$ (and also Z${}_{2}$ in Figure 3e) binds with the DNA D to form the complex C. Please note that for the model in Figure 3e, the conservation law becomes $D_{{\mathrm{tot}}_{j}}=D_{j}+C_{j_{1}}+C_{j_{2}}$, where $C_{j_{1}}$ is the complex formed by the binding of $Z_{1}$ with D and $C_{j_{2}}$ the one coming from the binding of $Z_{2}$ with D. The coefficient $k_{3_{j}}$ is the dissociation constant of the reversible binding reactions and is equal to $k_{3_{j}}^{-}\;/\;k_{3_{j}}^{+}$, where $k_{3}^{+}$ and $k_{3}^{-}$ are the association and dissociation rate constants, respectively. The presence of the downstream systems modifies the conservation laws of $I_{{\mathrm{tot}}_{1}}$ and $I_{{\mathrm{tot}}_{2}}$. These become $I_{{\mathrm{tot}}_{1}}=I_{1}+M_{1}+Z_{1}+\sum_{j=1}^{Q}C_{j}$ and $I_{{\mathrm{tot}}_{2}}=I_{2}+M_{2}+Z_{2}+\sum_{j=1}^{N-Q}C_{j}$, where *Q* is the number of upstream systems connected to the first upstream system, that in the second and in the fourth model is equal to *N*, while in the last one we have 0≤Q≤N. Thus, supposing no $M_{1}$, $M_{2}$, $Z_{1}$, $Z_{2}$, $C_{j}$ present in the system before $t_{0}$, we can write $I_{{\mathrm{tot}}_{1}}\left(t_{0}\right)=I_{1}\left(t_{0}\right)$ and $I_{{\mathrm{tot}}_{2}}\left(t_{0}\right)=I_{2}\left(t_{0}\right)$.

## 5. Results: Communication Performance Evaluation

In this section, we present the results obtained supposing a low and a high molecular count regime. Please note that due to the complexity of the calculations, not for all the case studies we determine an analytical formula of the MI. Nevertheless, we discuss whether retroactivity causes a reduction of the communication performance in all the considered scenarios.

### 5.1. Low Molecular Counts

For all the signaling system models, we make the same hypotheses on the values of $E_{\mathrm{tot}}$, $D_{{\mathrm{tot}}_{j}}$ and on the number of input symbols $n_{I_{1}}$, $n_{I_{2}}$, and their corresponding $I_{1}\left(t_{0}\right)$, $I_{2}\left(t_{0}\right)$ to compute the steady state solution of the CME. We choose $n_{I_{1}}=n_{I_{2}}=2$. This means that both the upstream systems have two available input symbols, one composed by 0 molecules of I ($I\left(t_{0}\right)=0$, no transmission) while the second composed by one molecule of I ($I\left(t_{0}\right)=1$). This corresponds to a Concentration On-Off Keying modulation [55]. This choice allows the explicit enumeration of all the microstates of the system, thus the analytical investigation of the role of retroactivity in the information exchange between $I_{1}\left(t_{0}\right)$ and $Z_{1}\left(t_{s}\right)$, where $t_{s}$ stands for steady state time. Accordingly, we also set $E_{\mathrm{tot}}$, $D_{{\mathrm{tot}}_{j}}=1$.

By solving the CME to obtain the necessary pmfs and by substituting *X* with $I_{1}\left(t_{0}\right)$ and *Y* with $Z_{1}\left(t_{s}\right)$, it is possible to show (see the derivations in the Appendix A) that the formula of the MI we obtain as difference of entropies *H* in the five considered cases is the same, corresponding to

(1) $$\begin{array}{cc} \; & I\left(I_{1}\left(t_{0}\right),Z_{1}\left(t_{s}\right)\right)=H\left(I_{1}\left(t_{0}\right)\right)-H\left(I_{1}\left(t_{0}\right)|Z_{1}\left(t_{s}\right)\right)\\ & =\log_{e}\left(P_{0_{1}}^{\left(-P_{0_{1}}\right)}P_{1_{1}}^{\left(-P_{1_{1}}\right)}\right)-\log_{e}\left(\left({\left(\frac{P_{0_{1}}}{P_{0_{1}}+AP_{1_{1}}}\right)}^{\left(-P_{0_{1}}\right)}\right)\cdot \left({\left(\frac{AP_{1_{1}}}{P_{0_{1}}+AP_{1_{1}}}\right)}^{\left(-AP_{1_{1}}\right)}\right)\right) \end{array}$$

nat/symbol, where $P_{0_{1}}$ and $P_{1_{1}}$ are the probabilities of the two possible input symbols $I_{1}\left(t_{0}\right)=0$ and $I_{1}\left(t_{0}\right)=1$. The term *A* is a constant depending on the coefficients of the chemical reactions of the upstream and downstream systems, and on the probabilities $P_{0_{2}}$ and $P_{1_{2}}$ of the input symbols of the second upstream system in the two MIMO models.

**Isolated SISO.** In the first case, when the model is composed by a single isolated SISO signaling system (Figure 3a), we obtain

(2) $$A=A_{0}=\frac{\left(1+k_{0}\right)c_{2}}{c_{1}+\left(1+k_{0}\right)c_{2}},$$

where $k_{0}=\left(k_{0}^{-}\Omega \right)\;/\;k_{0}^{+}$, Ω being the volume in which the reactions take place, as detailed in the Appendix A. This shows that the probability of the output $Z_{1}\left(t_{s}\right)$ is affected by the presence of the reaction $\begin{array}{c} Z_{1}\overset{\;c_{1}\;}{\to }I_{1} \end{array}$ that transforms the output protein $Z_{1}$ in the input $I_{1}$, not allowing the complete consumption of $I_{1}$ at steady state.

**SISO with*****N*****downstream targets.** The term *A* in the second system (Figure 3b) captures the impact of retroactivity on the information exchange due to *N* downstream systems. In this case, the formula of *A* is affected by the coefficients of the reactions connecting the upstream and the downstream systems. We obtain

(3) $$A=A_{N}=1-\frac{c_{1}}{\left(1+k_{0}\right)c_{2}+\left(1+\sum_{j=1}^{N}\left(1\;/\;k_{3_{j}}\right)\right)c_{1}},$$

where $k_{3_{j}}=\left(k_{3_{j}}^{-}\Omega \right)\;/\;k_{3_{j}}^{+}$. Please note that if we remove the downstream systems effect $\sum_{j=1}^{N}\left(1\;/\;k_{3_{j}}\right)$, we return to $A_{0}$. This system captures the impact of retroactivity on the information exchange performance in a generic biomolecular signaling BC. If we assume that all the *N* downstream systems have the same dissociation constant $k_{3}$, we can rewrite $A_{N}=1-\frac{c_{1}}{\left(1+k_{0}\right)c_{2}+\left(1+N\;/\;k_{3}\right)c_{1}}$. We observe that, for N→∞, $A_{N}$ is equal to 1. By substituting A=1 in (1), we obtain that $I\left(I_{1}\left(t_{0}\right),Z_{1}\left(t_{s}\right)\right)=0$, i.e., $I_{1}\left(t_{0}\right)$, $Z_{1}\left(t_{s}\right)$ are completely independent. This is a coherent result if we note that, for infinite downstream systems, the probability of the number of free molecules of $Z_{1}\left(t_{s}\right)$ being different from 0 tends to 0, independently of the value of $I_{1}\left(t_{0}\right)$.

We also note that, for $c_{2}=0$, i.e., if the upstream system is not cyclic, $A_{0}$ becomes 0, leading to $I\left(I_{1}\left(t_{0}\right),Z_{1}\left(t_{s}\right)\right)=\log_{e}\left(P_{0_{1}}^{\left(-P_{0_{1}}\right)}P_{1_{1}}^{\left(-P_{1_{1}}\right)}\right)$. This corresponds to having the MI equal to the entropy of $I_{1}\left(t_{0}\right)$. Thus, in this case $I_{1}\left(t_{0}\right)$ and $Z_{1}\left(t_{s}\right)$ are fully dependent. On the contrary, removing the cycle in the upstream system results in $A_{N}=1-\frac{c_{1}}{\left(1+\sum_{j=1}^{N}\left(1\;/\;k_{3_{j}}\right)\right)c_{1}}$. This is because the load given by the connected downstream systems still has an impact on the information exchange between $I_{1}$ and $Z_{1}$ by subtracting free $Z_{1}$ (i.e., the output message) from the system environment. If $c_{2}\to \infty$ we obtain $A=A_{0}=A_{N}=1$, thus the MI becomes 0, meaning that the input and the output are completely independent. In fact, for $c_{2}\to \infty$, the produced $Z_{1}$ is immediately transformed into $I_{1}$, so that the number of free molecules $Z_{1}\left(t_{s}\right)$ is equal to 0 for all values of $I_{1}\left(t_{0}\right)$. In general, the higher the *A*, the lower the communication exchange performance measured via (1).

**Isolated MIMO.** The formula of *A* for the third model (Figure 3c) is influenced by the presence of the second upstream system

(4) $$A=A_{0,\;\mathrm{MIMO}}=A_{0}P_{0_{2}}+BP_{1_{2}},$$

where $A_{0}$ comes from (2), and *B* is a constant depending on the chemical reaction rates as follows. Supposing all equal catalytic rates $c_{1}=c_{2}=c$ and supposing binding/unbinding rates $k_{0}^{+},k_{0}^{-}\gg c$, we can write *B* as

(5) $$B=\frac{3{\left(k_{0}^{+}\right)}^{2}+{\left(k_{0}^{-}\right)}^{2}+4k_{0}^{-}k_{0}^{+}+3k_{0}^{-}c+6k_{0}^{+}c}{5{\left(k_{0}^{+}\right)}^{2}+{\left(k_{0}^{-}\right)}^{2}+5k_{0}^{-}k_{0}^{+}+3k_{0}^{-}c+8k_{0}^{+}c},$$

where $k_{0}^{+}=k_{0}^{+}\;/\;\Omega$ (see Appendix A). If $P_{1_{2}}=0$ and by consequence $P_{0_{2}}=1$, we obtain $A_{0,\;\mathrm{MIMO}}=A_{0}$, thus we return to the single isolated SISO model. We demonstrated via Wolfram Mathematica^®^ software that $A_{0}P_{0_{2}}+BP_{1_{2}}\geq A_{0}$ is always true, for every value of $P_{0_{2}}$ and $P_{1_{2}}$, with the constraint that their sum should always be 1 and they cannot assume negative values. Thus, since $A_{0,\;\mathrm{MIMO}}$ is always greater than $A_{0}$, the MI in (1) is always lower in the case of isolated MIMO rather than in the case of isolated SISO. As in digital communication, also in MC, the communication performance of a MIMO system is worse than the ones of a SISO system given the same boundary conditions.

**MIMO with*****N*****downstream targets.** Likewise, the formula of *A* for the fourth system (Figure 3d) becomes

(6) $$A=A_{N,\;\mathrm{MIMO}}=A_{N}P_{0_{2}}+GP_{1_{2}}$$

where $A_{N}$ is (3) and *G* depends on the rates of the chemical reactions. The analytical formula of *G* cannot be easily expressed in closed form, although it is worth noticing that it depends not only on the reaction rates of the upstream, but also on the binding/unbinding rates of the downstream systems. This is a key difference with respect to *B* (5). Under some assumptions, i.e., all equal (un)binding rates $k^{+},k^{-}$, all equal catalytic rates c, and $k^{+},k^{-}\gg c$, we obtain via Wolfram Mathematica^®^ software G>B and $A_{N}>A_{0}$. From these, it follows $A_{N,\;\mathrm{MIMO}}>A_{0,\;\mathrm{MIMO}}\;\left(4\right)$, i.e., when the output $Z_{1}$ of the MIMO upstream system is connected with *N* downstream systems, the MI lowers.

In this case, when $P_{1_{2}}=0$, we return to the second model, i.e., the SISO upstream system and BC. Notwithstanding this, we would like to understand if $A_{N,\;\mathrm{MIMO}}$ is always greater than $A_{N}$, i.e., if the presence of a second upstream system lowers the MI. To do that, we set the inequality $A_{N,\;\mathrm{MIMO}}\geq A_{N}$, and we solve it with respect to *G*. Specifically

(7) $$\begin{array}{cc} \; & A_{N}P_{0_{2}}+GP_{1_{2}}\geq A_{N}\\ \Rightarrow \; & A_{N}\left(P_{0_{2}}-1\right)-GA_{N}\left(P_{0_{2}}-1\right)\geq 0\\ \Rightarrow \; & G\geq A_{N}. \end{array}$$

From (7), we observe that it would be theoretically possible that for some values of *G* (i.e., for $G<A_{N}$), $I\left(I_{1}\left(t_{0}\right),Z_{1}\left(t_{s}\right)\right)$ of the system in Figure 3d becomes higher than the one in Figure 3b. This would imply that a MIMO upstream system binding with the same number *N* of downstream as the single SISO upstream can mitigate the impact of retroactivity on $I\left(I_{1}\left(t_{0}\right),Z_{1}\left(t_{s}\right)\right)$.

**Two isolated SISO with MAC.** The expression of *A* in the model of Figure 3e is noteworthy. In fact, although there are two upstream systems in the environment, they are completely independent, meaning that the presence of the second does not have any influence on the communication performance of the first one. Since the two systems share the binding with the same *N* downstream systems, *A* becomes

(8) $$A=A_{Q,\;\mathrm{MAC}}=1-\frac{c_{1}}{\left(1+k_{0}\right)c_{2}+\left(1+\sum_{j=1}^{Q}\left(1\;/\;k_{3_{j}}\right)\right)c_{1}}.$$

The number of downstream systems binding with the second upstream is always complementary to *Q*, and it is equal to N−Q. Supposing that the two upstream systems emit the same number of molecules, i.e., $Z_{1}$, $Z_{2}$, then on average Q=N/2. If Q=0, then (8) becomes (2), while in the opposite case, when Q=N, then $A_{Q,\;\mathrm{MAC}}=A_{N}$ (3). From this, we note that the fifth model represents an improvement in relation to the communication performance with respect to the model of Figure 3b. In fact, since the second upstream system binds with some of the available downstream systems, the retroactivity observed at the first upstream system is lowered.

**Study of A term via Z-channel capacity and capacity bounds.** Please note that regardless of the specific signaling system, each of these cases can be traced back to a Z-channel. In fact, the joint pmf $P\left(I_{1}\left(t_{0}\right),Z_{1}\left(t_{s}\right)\right)$ is for all the five models

(9) $$P\left(I_{1}\left(t_{0}\right),Z_{1}\left(t_{s}\right)\right)=\left[\begin{array}{cc} P_{0_{1}} & 0\\ AP_{1_{1}} & \left(1-A\right)P_{1_{1}} \end{array}\right],$$

where the rows represent the 0 and 1 possible values of $I_{1}\left(t_{0}\right)$ and the columns that of $Z_{1}\left(t_{s}\right)$ (see Appendix A for the exact derivation). From (9), it is clear that if $I_{1}\left(t_{0}\right)=0$, then $Z_{1}\left(t_{s}\right)$ is certainly equal to 0, while if $I_{1}\left(t_{0}\right)=1$, then $Z_{1}\left(t_{s}\right)$ is 1 with a certain probability $\left(1-A\right)$ and 0 with probability *A*.

**Figure 4:**
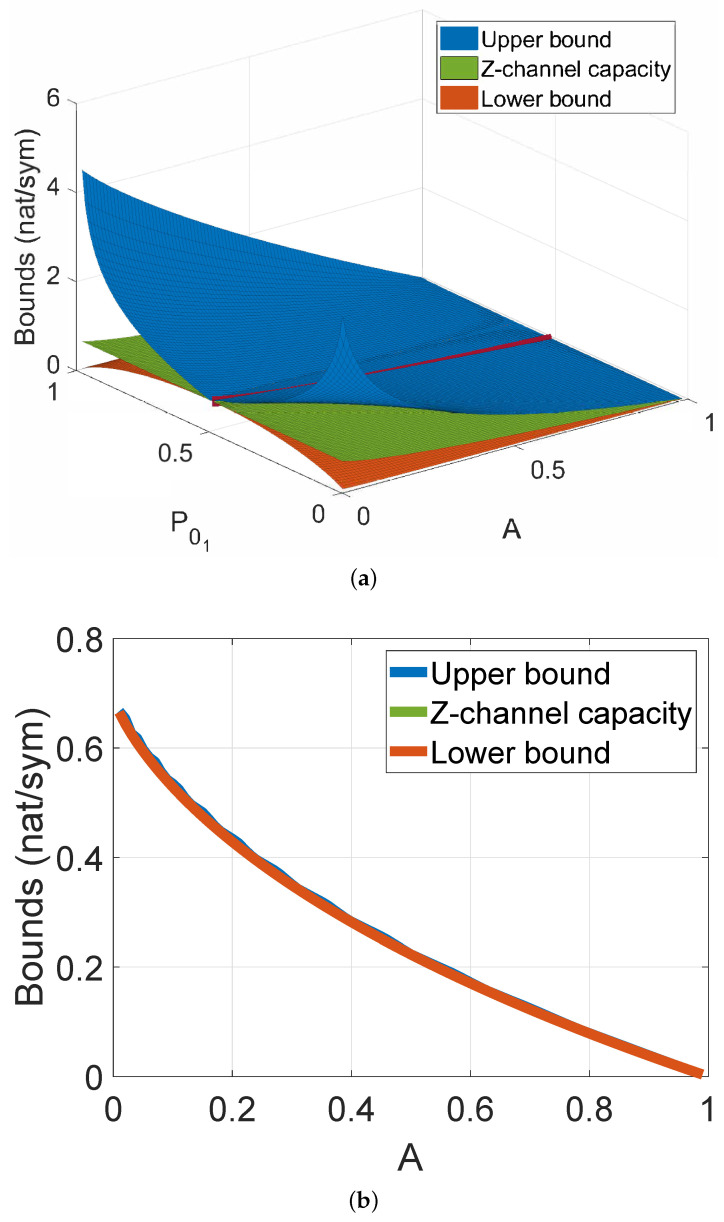
(**a**) Upper, lower bounds on the channel capacity, and Z-channel capacity for the four signaling systems, by varying $P_{0_{1}}$ and *A*. (**b**) Channel capacity bounds and Z-Channel capacity with respect to the valid range of *A* values.

For this reason, we validate our MI Formula (1), by plotting the Z-channel capacity [56], where the conditional probability $P\left(X=1|Y=0\right)$ in our case is to be substituted with *A*, and by varying the values of *A* and of $P_{0_{1}}$. Please note that since both *A* and $P_{0_{1}}$ are probabilities, the range of valid values that they can assume is included between 0 and 1. Furthermore, we plot on the same graph the MI we obtained (1). By doing this, as it can be observed from the graph, we are also finding a lower bound on the channel capacity for each $\left(A,P_{0_{1}}\right)$ and, for each *A*, what is the value of $P_{0_{1}}$ that maximize it, thus the value of the channel capacity. We plot also the upper bound on the channel capacity starting from the definition of MI as Kullback–Leibler divergence as described in [57] in 3D space. In this way, we note that the minimum of the upper bound, the Z-channel capacity, and the maximum of the lower bound for each value of *A* coincide (red line). Figure 4a is the visualization of what has just been described. Thanks to this 3D plotting, we are able to isolate the channel capacity value for each value of *A* (Figure 4b), that, as for each value of the MI, decreases as *A* increases, confirming our theoretical results.

### 5.2. High Molecular Counts

As mentioned in Section 3.2, the LNA describes the system through an ordinary differential equation and a linear (time-varying) stochastic differential Equation [48]. To mitigate the analytical complexity, when only a single upstream system is present, we reduce it to a one-step cyclic enzymatic reaction

$$\begin{array}{c} I_{1}+E\overset{\;c_{1}\;}{\to }E+Z_{1}\\ Z_{1}\overset{\;c_{2}\;}{\to }I_{1}, \end{array}$$

with conservation law $I_{{\mathrm{tot}}_{1}}=I_{1}+Z_{1}+\sum_{j=1}^{N}C_{j}$. Thus, in the case of isolated SISO system, the differential equation characterizing the variation of the mean of Z${}_{1}$ with time can be written as

(10) $$\frac{dZ_{1}}{dt}=c_{1}E\left(I_{{\mathrm{tot}}_{1}}-Z_{1}\right)-c_{2}Z_{1},$$

where $\sum_{j=1}^{N}C_{j}$ is equal to 0 (no downstream systems). Then, the mean of the Gaussian distribution characterizing Z${}_{1}\left(t_{s}\right)$ can be found easily by setting (10) equal to 0 and by solving the resulting equation with respect to Z${}_{1}$

(11) $$\mu_{0}=\frac{c_{1}EI_{{\mathrm{tot}}_{1}}}{c_{2}+c_{1}E}.$$

The variance $\sigma_{0}^{2}$ of the same distribution is obtained by solving the Lyapunov equation

(12) $$\begin{array}{cc} 2\left(-c_{1}E-c_{2}\right)\sigma_{0}^{2} & =-\frac{1}{\Omega }\left(c_{1}E\left(I_{{\mathrm{tot}}_{1}}-\frac{c_{1}EI_{{\mathrm{tot}}_{1}}}{c_{2}+c_{1}E}\right)+c_{2}\frac{c_{1}EI_{{\mathrm{tot}}_{1}}}{c_{2}+c_{1}E}\right)\\ \Rightarrow \sigma_{0}^{2} & =\frac{c_{1}EI_{{\mathrm{tot}}_{1}}c_{2}}{\Omega {\left(c_{1}E+c_{2}\right)}^{2}}, \end{array}$$

where Ω is the volume of the environment (i.e., usually a cell). Then, remembering that the LNA supposes $\sigma_{noise}^{2}=1$, it is straightforward deriving the channel capacity by substituting (12) in its well-known formula [49].

Supposing equal dissociation constant $k_{3}$ for all the downstream systems, their characterizing differential equation is

(13) $$\frac{dC_{j}}{dt}=k_{3}^{+}Z_{1}D_{j}-k_{3}^{-}C_{j}=k_{3}^{+}Z_{1}\left(D_{{\mathrm{tot}}_{j}}-C_{j}\right)-k_{3}^{-}C_{j},$$

where $Z_{1}$ is to be substituted with $Z_{2}$ when the second upstream system connects with the *j*th downstream one. Thus, the steady state value of $C_{j}$ when connected to the first upstream system is

(14) $$C_{j}=\frac{Z_{1}D_{{\mathrm{tot}}_{j}}}{k_{3}+Z_{1}}.$$

Then, for the second model (SISO with *N* downstream systems, Figure 3b) we obtain

(15) $$\begin{array}{cc} \frac{dZ_{1}}{dt} & =c_{1}E\left(I_{{\mathrm{tot}}_{1}}-Z_{1}-\sum_{j=1}^{N}C_{j}\right)+k_{3}^{-}\sum_{j=1}^{N}C_{j}-c_{2}Z_{1}-k_{3}^{+}Z_{1}\sum_{j=1}^{N}\left(D_{{\mathrm{tot}}_{j}}-C_{j}\right). \end{array}$$

To obtain the mean value of $Z\left(t_{s}\right)$ distribution, we should substitute (14) in (15). Then, supposing that the *N* downstream systems have equal conservation law $D_{\mathrm{tot}}=D+C$, we obtain $\mu_{N}=\mu_{0}$. This means that in the high molecular count regime the mean of the $Z\left(t_{s}\right)$ distribution is not impacted by the presence of the downstream systems. Due to the higher computational complexity, we do not provide the formula of the variance $\sigma_{N}^{2}$ in this case. However, to determine it one would have to solve the Lyapunov equation for this system with respect to $\sigma_{N}^{2}$.

A two-step enzymatic reaction for the upstream systems is intrinsic of MIMO models. Thus, we reuse the chemical reactions modeling of the low molecular counts regime. For this reason, the system of differential equations characterizing the third model (Figure 3c) is

(16) $$\left\{\begin{array}{c} \frac{dZ_{1}}{dt}=c_{1}M_{1}-c_{2}Z_{1}\\ \frac{dM_{1}}{dt}=k_{0}^{+}EI_{1}-\left(k_{0}^{-}+c_{1}\right)M_{1}\\ \frac{dZ_{2}}{\mathrm{dt}}=c_{1}M_{2}-c_{2}Z_{2}\\ \frac{dM_{2}}{dt}=k_{0}^{+}EI_{2}-\left(k_{0}^{-}+c_{1}\right)M_{2}. \end{array}\right.$$

From the conservation laws, $I_{1}=I_{{\mathrm{tot}}_{1}}-M_{1}-Z_{1}$, $I_{2}=I_{{\mathrm{tot}}_{2}}-M_{2}-Z_{2}$, and $E=E_{\mathrm{tot}}-M_{1}-M_{2}$.

Similarly, the system in Figure 3d can be written as

(17) $$\left\{\begin{array}{c} \frac{dZ_{1}}{dt}=c_{1}M_{1}+k_{3}^{-}\sum_{j=1}^{N}C_{j}-Z_{1}\left(c_{2}+k_{3}^{+}\sum_{j=1}^{N}\left(D_{{\mathrm{tot}}_{j}}-C_{j}\right)\right)\\ \frac{dM_{1}}{dt}=k_{0}^{+}\left(E_{\mathrm{tot}}-M_{1}-M_{2}\right)\left(I_{{\mathrm{tot}}_{1}}-M_{1}-Z_{1}-\sum_{j=1}^{N}C_{j}\right)-\left(k_{0}^{-}+c_{1}\right)M_{1}\\ \frac{dZ_{2}}{dt}=c_{1}M_{2}-c_{2}Z_{2}\\ \frac{dM_{2}}{dt}=k_{0}^{+}\left(E_{\mathrm{tot}}-M_{1}-M_{2}\right)\left(I_{{\mathrm{tot}}_{2}}-M_{2}-Z_{2}\right)-\left(k_{0}^{-}+c_{1}\right)M_{2}, \end{array}\right.$$

where $C_{j}$ is to be substituted with (14) in the first two differential equations.

Both (16) and (17), after being set equal to 0, are to be solved with respect to $Z_{1}$ to obtain the mean value of the Gaussian-distributed $Z\left(t_{s}\right)$. We do not report here these results and the Lyapunov equation that would give the variance in these cases, due to their analytical complexity.

The differential equation characterizing $Z_{1}$ of the fifth system (Figure 3e) is analogous to (15), considering the number of downstream systems 0≤Q≤N and remembering $D_{\mathrm{tot}}=D+C_{j_{1}}+C_{j_{2}}$. The same analytical complexity applies for the calculation of the variance.

## 6. Conclusions

In this paper, we evaluate the effect of retroactivity on communication performance of different signaling systems. More specifically, we considered five different biomolecular circuits with analogies to some well-known digital communication system models. For each of them, we evaluated the analytical formula of the MI between the input at time 0 (i.e., the beginning of the transmission of a symbol) and the output evaluated at steady state. The steady state assumption implies the absence of intersymbol interference and the mitigation of the transient behavior of the system.

Our results show that retroactivity has a negative impact on the communication performance, by reducing the MI as the number of downstream systems increases. We note also how the effect of retroactivity can be mitigated by the presence of a second independent upstream system, that connects with the same pool of downstream systems. The same behavior occurs for all the five systems both in the high and in the low molecular count regime.

The analytical computation of the Gaussian distributed output in the high molecular count regime for the remaining cases, as well as a biological interpretation of the *A* constant in the low molecular count regime are left for future work.

Nevertheless, we can make some hypotheses on the analytical results in the high molecular counts. For the SISO with *N* downstream targets model, given that in the low molecular count regime the MI of this system is lower than the one in the isolated SISO case, and by remembering the AWGN channel capacity formula [49], we can speculate that $\sigma_{N}^{2}=\sigma_{0}^{2}-K$, where *K* is a positive constant. If true, this would imply a lower channel capacity value than in the isolated SISO scenario. Regarding the two isolated SISO with MAC model, since the number of downstream systems connected to $Z_{1}$ is less or equal than the one of SISO with *N* downstream targets case, we speculate that in this case the variance $\sigma_{Q,\;\mathrm{MAC}}^{2}$ would be greater than $\sigma_{N}^{2}$.

Future work will develop in multiple directions. First of all we are interested in linking the effect of retroactivity on cell behavior, in the same line of [58]. Another line of research is that of comparing strategies to quantify the MI and information transmission and observe if the quantification of the impact of retroactivity holds. Furthermore, it would be worth generalizing our method to evaluate the impact of retroactivity in the case of more complex systems, as for example in the case of a set of non linear differential equations.

## Figures and Tables

**Figure A1:**
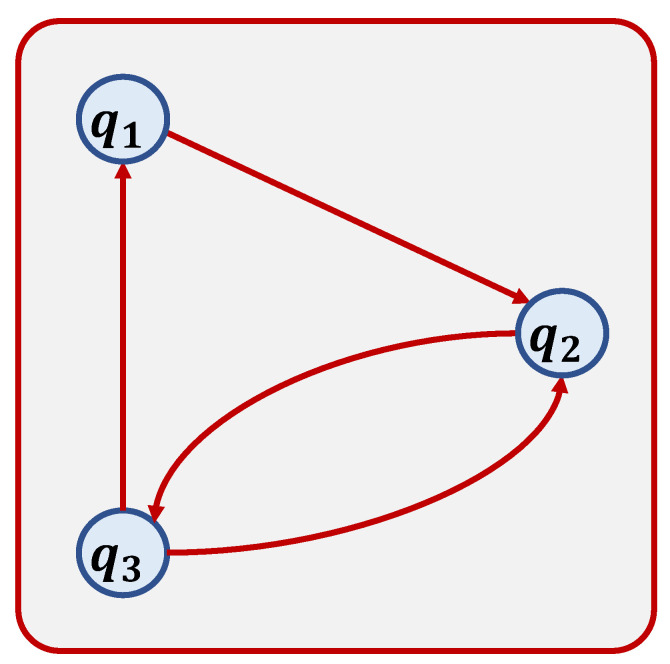
Transitions between microstates for the isolated SISO system model.

## Data Availability

Not applicable.

## Appendix A

This appendix is devoted to the derivation of the expression of the MI (1). In all the five considered scenarios, the first step to perform is that of recalling the marginal distribution of the input

(A1) $$P\left(I_{1}\left(t_{0}\right)\right)=\left[\begin{array}{c} P_{0_{1}}\\ P_{1_{1}} \end{array}\right],$$

and that of evaluating $P\left(I_{1}\left(t_{0}\right)|Z_{1}\left(t_{s}\right)\right)$. To do this, it is necessary to solve the CME. The first step consists of enumerating all the possible microstates of the biomolecular system. Clearly, for the same number of molecules, as the number of species present in the system increases, also the number of possible microstates becomes higher. In our work, we consider $n_{I_{1}}=n_{I_{2}}=2$, thus $I_{{\mathrm{tot}}_{1}}\leq 1$ and $I_{{\mathrm{tot}}_{2}}\leq 1$. Furthermore, we set $E_{\mathrm{tot}}=D_{{\mathrm{tot}}_{j}}=1$. The following Table A1 and Table A2 resume all the possible microstates $q_{i}$ for each considered system when $I_{{\mathrm{tot}}_{1}}=0$, $I_{{\mathrm{tot}}_{2}}=1$ and $I_{{\mathrm{tot}}_{1}}=1$, $I_{{\mathrm{tot}}_{2}}=1$, respectively. Please note that we consider $I_{{\mathrm{tot}}_{2}}=1$ because we are interested in the value of the MI in the presence of the molecule emitted by the second upstream system. For simplicity, we suppose to have only one downstream system, i.e., j=1.

**Table A1:** Microstates of the systems when $I_{{\mathrm{tot}}_{1}}=0$, $I_{{\mathrm{tot}}_{2}}=1$.

System Model	State	$M_{1}$	$I_{1}$	$Z_{1}$	$C_{1}$	$M_{2}$	$I_{2}$	$Z_{2}$	$C_{2}$	E	$E_{2}$	D
Isolated SISO	$q_{1}$	0	0	0	0	0	0	0	0	1	0	0
SISO + downst.	$q_{1}$	0	0	0	0	0	0	0	0	1	0	1
Isolated MIMO	$q_{1}$	0	0	0	0	0	0	1	0	1	0	0
	$q_{2}$	0	0	0	0	0	1	0	0	1	0	0
	$q_{3}$	0	0	0	0	1	0	0	0	0	0	0
MIMO with 1 downstream target	$q_{1}$	0	0	0	0	0	0	1	0	1	0	1
	$q_{2}$	0	0	0	0	0	1	0	0	1	0	1
	$q_{3}$	0	0	0	0	1	0	0	0	0	0	1
Two isolated SISO with MAC	$q_{1}$	0	0	0	0	0	0	0	1	0	1	0
	$q_{2}$	0	0	0	0	0	0	1	0	0	1	1
	$q_{3}$	0	0	0	0	0	1	0	0	0	1	1
	$q_{4}$	0	0	0	0	1	0	0	0	0	0	1

**Table A2:** Microstates of the systems when $I_{{\mathrm{tot}}_{1}}=1$, $I_{{\mathrm{tot}}_{2}}=1$.

System Model	State	$M_{1}$	$I_{1}$	$Z_{1}$	$C_{1}$	$M_{2}$	$I_{2}$	$Z_{2}$	$C_{2}$	E	$E_{2}$	D
Isolated SISO	$q_{1}$	0	0	1	0	0	0	0	0	1	0	0
	$q_{2}$	0	1	0	0	0	0	0	0	1	0	0
	$q_{3}$	1	0	0	0	0	0	0	0	0	0	0
SISO with 1 downstream target	$q_{1}$	0	0	0	1	0	0	0	0	1	0	0
	$q_{2}$	0	0	1	0	0	0	0	0	1	0	1
	$q_{3}$	0	1	0	0	0	0	0	0	1	0	1
	$q_{4}$	1	0	0	0	0	0	0	0	0	0	1
Isolated MIMO	$q_{1}$	0	0	1	0	0	0	1	0	1	0	0
	$q_{2}$	0	0	1	0	0	1	0	0	1	0	0
	$q_{3}$	0	0	1	0	1	0	0	0	0	0	0
	$q_{4}$	0	1	0	0	0	0	1	0	1	0	0
	$q_{5}$	0	1	0	0	0	1	0	0	1	0	0
	$q_{6}$	0	1	0	0	1	0	0	0	0	0	0
	$q_{7}$	1	0	0	0	0	1	0	0	0	0	0
	$q_{8}$	1	0	0	0	1	0	0	0	0	0	0
MIMO with 1 downstream target	$q_{1}$	0	0	0	1	0	0	1	0	1	0	0
	$q_{2}$	0	0	0	1	0	1	0	0	1	0	0
	$q_{3}$	0	0	0	1	1	0	0	0	0	0	0
	$q_{4}$	0	0	1	0	0	0	1	0	1	0	1
	$q_{5}$	0	0	1	0	0	1	0	0	1	0	1
	$q_{6}$	0	0	1	0	1	0	0	0	0	0	1
	$q_{7}$	0	1	0	0	0	0	1	0	1	0	1
	$q_{8}$	0	1	0	0	0	1	0	0	1	0	1
	$q_{9}$	0	1	0	0	1	0	0	0	0	0	1
	$q_{10}$	1	0	0	0	0	0	1	0	0	0	1
	$q_{11}$	1	0	0	0	0	1	0	0	0	0	1
Two isolated SISO with MAC	$q_{1}$	0	0	0	1	0	0	1	0	1	1	0
	$q_{2}$	0	0	0	1	0	1	0	0	1	1	0
	$q_{3}$	0	0	0	1	1	0	0	0	1	0	0
	$q_{4}$	0	0	1	0	0	0	0	1	1	1	0
	$q_{5}$	0	0	1	0	0	0	1	0	1	1	1
	$q_{6}$	0	0	1	0	0	1	0	0	1	1	1
	$q_{7}$	0	0	1	0	1	0	0	0	1	0	1
	$q_{8}$	0	1	0	0	0	0	0	1	1	1	0
	$q_{9}$	0	1	0	0	0	0	1	0	1	1	1
	$q_{10}$	0	1	0	0	0	1	0	0	1	1	1
	$q_{11}$	0	1	0	0	1	0	0	0	1	0	1
	$q_{12}$	1	0	0	0	0	0	0	1	0	1	0
	$q_{13}$	1	0	0	0	0	0	1	0	0	1	1
	$q_{14}$	1	0	0	0	0	1	0	0	0	1	1
	$q_{15}$	1	0	0	0	1	0	0	0	0	0	1

From now on, we report the mathematical steps only for the isolated SISO system, analogous results are obtained for the remaining models. Both in the case of $I_{{\mathrm{tot}}_{1}}=0$ and of $I_{{\mathrm{tot}}_{1}}=1$, the set of chemical reactions representing the biomolecular system can be rewritten as a set of transitions between the enumerated microstates, as depicted in Figure A1 for the isolated SISO system in the case of $I_{{\mathrm{tot}}_{1}}=1$. Clearly, since the case with $I_{{\mathrm{tot}}_{1}}=0$ is characterized only by one microstate, the system remains in $q_{1}$ with probability equal to 1 in each instant of time *t*, i.e., $P\left(q_{1},t\right)=1$. When $I_{{\mathrm{tot}}_{1}}=1$, and assuming that all reactions take place in a volume Ω, it is possible to use the propensity functions, i.e., the probability that a given reaction occurs in a sufficiently small amount of time, to obtain

$$\begin{array}{c} I_{1}+E\overset{\;k_{0}^{+}\;}{\to }M_{1}:\;q_{2}\to q_{3};\;a_{1}\left(q_{2}\right)=k_{0}^{+}=\left(k_{0}^{+}\;/\;\Omega \right)\\ I_{1}+E\overset{\;k_{0}^{-}\;}{\leftarrow }M_{1}:\;q_{3}\to q_{2};\;a_{2}\left(q_{3}\right)=k_{0}^{-}\\ M_{1}\overset{\;c_{1}\;}{\to }E+Z_{1}:\;q_{3}\to q_{1};\;a_{3}\left(q_{3}\right)=c_{1}\\ Z_{1}\overset{\;c_{2}\;}{\to }I_{1}:\;q_{1}\to q_{2};\;a_{4}\left(q_{1}\right)=c_{2}. \end{array}$$

Then, the CME can be written down using the propensity functions for each reaction. For the isolated SISO system it becomes

(A2) $$\frac{d}{dt}\left[\begin{array}{c} P\left(q_{1},t\right)\\ P\left(q_{2},t\right)\\ P\left(q_{3},t\right) \end{array}\right]=\left[\begin{array}{ccc} -c_{2} & 0 & c_{1}\\ c_{2} & -k_{0}^{+} & k_{0}^{-}\\ 0 & k_{0}^{+} & -k_{0}^{-}-c_{1} \end{array}\right]\left[\begin{array}{c} P\left(q_{1},t\right)\\ P\left(q_{2},t\right)\\ P\left(q_{3},t\right) \end{array}\right].$$

In turn, the steady state solution for the probabilities $P\left(q_{i},t_{s}\right)$ can be solved by setting $\dot{P}=0$, which yields

(A3) $$\begin{array}{cc} \; & P\left(q_{1},t_{s}\right)=\frac{k_{0}^{+}c_{1}}{k_{0}^{+}c_{1}+c_{2}\left(k_{0}^{-}+k_{0}^{+}+c_{1}\right)}\\ \; & P\left(q_{2},t_{s}\right)=\frac{c_{2}\left(k_{0}^{-}+c_{1}\right)}{k_{0}^{+}c_{1}+c_{2}\left(k_{0}^{-}+k_{0}^{+}+c_{1}\right)}\\ \; & P\left(q_{3},t_{s}\right)=\frac{k_{0}^{+}c_{2}}{k_{0}^{+}c_{1}+c_{2}\left(k_{0}^{-}+k_{0}^{+}+c_{1}\right)}. \end{array}$$

Note that these probabilities can be seen as the joint pmf $P\left(I_{1}\left(t_{s}\right),Z_{1}\left(t_{s}\right)\right)$, where

(A4) $$\begin{array}{cc} \; & P\left(q_{1},t_{s}\right)=P\left(I_{1}\left(t_{s}\right)=0,Z_{1}\left(t_{s}\right)=1\right),\\ \; & P\left(q_{2},t_{s}\right)=P\left(I_{1}\left(t_{s}\right)=1,Z_{1}\left(t_{s}\right)=0\right),\\ \; & P\left(q_{3},t_{s}\right)=P\left(I_{1}\left(t_{s}\right)=0,Z_{1}\left(t_{s}\right)=0\right). \end{array}$$

Thus, if we multiply $P\left(q_{1},t\right)=1$ by $P_{0_{1}}$ and (A4) by $P_{1_{1}}$, we easily obtain the joint probability $P\left(I_{1}\left(t_{s}\right),Z_{1}\left(t_{s}\right),I_{1}\left(t_{0}\right)\right)$. By marginalizing over $I_{1}\left(t_{s}\right)$, we finally obtain

(A5) $$\begin{array}{cc} \; & P\left(I_{1}\left(t_{0}\right),Z_{1}\left(t_{s}\right)\right)=\left[\begin{array}{cc} P_{0_{1}} & 0\\ \frac{c_{2}\left(k_{0}^{-}+k_{0}^{+}+c_{1}\right)}{k_{0}^{+}c_{1}+c_{2}\left(k_{0}^{-}+k_{0}^{+}+c_{1}\right)}P_{1_{1}} & \left(1-\frac{c_{2}\left(k_{0}^{-}+k_{0}^{+}+c_{1}\right)}{k_{0}^{+}c_{1}+c_{2}\left(k_{0}^{-}+k_{0}^{+}+c_{1}\right)}\right)P_{1_{1}} \end{array}\right], \end{array}$$

where the rows represent the possible values assumed by $I_{1}\left(t_{0}\right)$, i.e., 0 and 1, and the columns the ones assumed by $Z_{1}\left(t_{s}\right)$.

By remembering that the dissociation constant is defined as $k_{0}=k_{0}^{-}\;/\;k_{0}^{+}$, we introduce $k_{0}=k_{0}^{-}\;/\;k_{0}^{+}$. Furthermore, we hypothesize $c_{1},c_{2}\ll k_{0}^{-},k_{0}^{+}$. Then, we rewrite (A5) as

(A6) $$P\left(I_{1}\left(t_{0}\right),Z_{1}\left(t_{s}\right)\right)=\left[\begin{array}{cc} P_{0_{1}} & 0\\ \frac{\left(1+k_{0}\right)c_{2}}{c_{1}+\left(1+k_{0}\right)c_{2}}P_{1_{1}} & \left(1-\frac{\left(1+k_{0}\right)c_{2}}{c_{1}+\left(1+k_{0}\right)c_{2}}\right)P_{1_{1}} \end{array}\right],$$

where $\frac{\left(1+k_{0}\right)c_{2}}{c_{1}+\left(1+k_{0}\right)c_{2}}=A$.

Having $P\left(I_{1}\left(t_{0}\right),Z_{1}\left(t_{s}\right)\right)$, it is straightforward to obtain $P\left(Z_{1}\left(t_{s}\right)\right)$ by marginalizing over $I_{1}\left(t_{0}\right)$, and then

(A7) $$P\left(I_{1}\left(t_{0}\right)|Z_{1}\left(t_{s}\right)\right)=\frac{P\left(I_{1}\left(t_{0}\right),Z_{1}\left(t_{s}\right)\right)}{P\left(Z_{1}\left(t_{s}\right)\right)}=\left[\begin{array}{cc} \frac{P_{0_{1}}}{P_{0_{1}}+AP_{1_{1}}} & 0\\ \frac{AP_{1_{1}}}{P_{0_{1}}+AP_{1_{1}}} & 1 \end{array}\right].$$

The entropies are now easily evaluated

(A8) $$\begin{array}{cc} H\left(I_{1}\left(t_{0}\right)\right) & =-\sum_{j=0}^{1}P\left(I_{1_{j}}\left(t_{0}\right)\right)\log\left(P\left(I_{1_{j}}\left(t_{0}\right)\right)\right)=\log_{e}\left(P_{0_{1}}^{\left(-P_{0_{1}}\right)}P_{1_{1}}^{\left(-P_{1_{1}}\right)}\right), \end{array}$$

and

(A9) $$\begin{array}{cc} \; & H\left(I_{1}\left(t_{0}\right)|Z_{1}\left(t_{s}\right)\right)\\ \; & =-\sum_{i=0}^{1}\sum_{j=0}^{1}P\left(Z_{1_{i}}\left(t_{s}\right)\right)P\left(I_{1_{j}}\left(t_{0}\right)|Z_{1_{i}}\left(t_{s}\right)\right)\log_{e}\left(P\left(I_{1_{j}}\left(t_{0}\right)|Z_{1_{i}}\left(t_{s}\right)\right)\right)\\ & =\log_{e}\left(\left({\left(\frac{P_{0_{1}}}{P_{0_{1}}+AP_{1_{1}}}\right)}^{\left(-P_{0_{1}}\right)}\right)\cdot \left({\left(\frac{AP_{1_{1}}}{P_{0_{1}}+AP_{1_{1}}}\right)}^{\left(-AP_{1_{1}}\right)}\right)\right). \end{array}$$

In turn, the MI is

(A10) $$\begin{array}{cc} \; & I\left(I_{1}\left(t_{0}\right),Z_{1}\left(t_{s}\right)\right)=H\left(I_{1}\left(t_{0}\right)\right)-H\left(I_{1}\left(t_{0}\right)|Z_{1}\left(t_{s}\right)\right)\\ \; & =\log_{e}\left(P_{0_{1}}^{\left(-P_{0_{1}}\right)}P_{1_{1}}^{\left(-P_{1_{1}}\right)}\right)-\log_{e}\left(\left({\left(\frac{P_{0_{1}}}{P_{0_{1}}+AP_{1_{1}}}\right)}^{\left(-P_{0_{1}}\right)}\right)\cdot \left({\left(\frac{AP_{1_{1}}}{P_{0_{1}}+AP_{1_{1}}}\right)}^{\left(-AP_{1_{1}}\right)}\right)\right) \end{array}$$

nat/symbol.
